# Pattern of traumatised anterior teeth among adult Nigerians and complications from late presentation

**DOI:** 10.1186/s13104-016-1871-3

**Published:** 2016-02-06

**Authors:** Joan Emien Enabulele, Adeleke O. Oginni, Matthew A. Sede, Fadekemi O. Oginni

**Affiliations:** Department of Restorative Dentistry, University of Benin, Benin City, 300001 Edo State Nigeria; Department of Restorative Dentistry, Obafemi Awolowo University, Ife, Osun State Nigeria; Department of Oral and Maxillofacial Surgery, Obafemi Awolowo University, Ife, Osun State Nigeria

## Abstract

**Background:**

The objectives of this study were to determine the prevalence and pattern of traumatic dental injury (TDI) among adults presenting in a tertiary health care facility in Nigeria, the time interval between injury and presentation in the hospital, the reasons for seeking treatment, and the complications arising due to late presentation.

**Methods:**

Information obtained from history taking and clinical examination included Patient’s demographics, the when, where, and how of the injury, previous history of trauma, time elapsed between injury and presentation at the hospital, reason for seeking treatment, tooth/teeth involved, sensibility, tenderness to percussion, mobility, presence of discolouration, swelling, sinus tract, and mobility. Radiographic findings such as periapical radiolucency, pulp canal obliteration, and root resorption were also documented. TDI was classified using the Andreasen’s classification.

**Results:**

Out of the 2645 adult patients that attended the outpatient clinic, 184 Presented with TDI giving a prevalence of 6.96 %. Their age range and mean age were 17–69 years and 30.6 ± 11.2 years respectively. Falls accounted for most (26.4 %) of the trauma to anterior teeth followed by motor cycle accidents (18.4 %) and domestic accidents (12.9 %), while opening bottle cork accounted for 1.2 %. The most common type of injury was enamel-dentine fracture accounting for 28.8 %, followed by complicated crown fracture 18.6 %, and avulsion 11.7 %. The maxillary central incisors were the most commonly affected followed by the maxillary lateral incisors and canines. More than half (51.5 %) of the patients presented in the hospital more than 1 year after injury. Majority of patients in the age groups ≤20 and 51–60 sought treatment because of pain, while more of those in the age groups 21–30 and 31–40 sought treatment because of aesthetic considerations. Seventy three (45.3 %) of the patients presented with complications involving 138 teeth. Majority of the complications were in teeth with enamel-dentine fracture (93.4 %), followed by concussion injury (55.6 %).

**Conclusions:**

The prevalence of TDI in this study falls within previously reported figures. However, the high number of teeth developing complications may have resulted from late presentation for care.

## Background

Nigeria, according to World Bank country classification is a low income developing country, besieged with diverse developmental problems. In Nigeria, the high spate of violence, increased participation in sporting activities and road traffic crashes have contributed immensely to traumatic dental injuries (TDI), thus making it an important oral health problem. Trauma to anterior teeth can cause several aesthetic and functional alterations, such as fractures, tooth discolouration, tooth mobility, toothache and tooth loss [1]. Such alterations may have a considerable impact on the quality of life of affected individuals [2]. It has been proven that trauma to anterior teeth can lead to social and psychological limitations, which may manifest as feeling embarrassed to smile, having difficulty with social relationships, inability to maintain a balanced emotional state, which in turn, provokes a state of irritation, dietary restrictions and difficulty in cleaning the teeth [3].

Traumatized anterior teeth are a common presentation in clinical dental practice. The magnitude of this problem has been reported in different parts of the world based on the prevalence of dental trauma during childhood and adolescence with the causes of TDI varying with age group. It has been reported that during childhood the main causes of TDI are falls [4]. Among adolescents, trauma to anterior teeth is mainly due to sports related accidents [5], while at the end of adolescence and beginning of adulthood, car accidents are the most prevalent causes [6].Trauma related to violence has also been reported in 9–12 year olds [7].

The most frequently traumatized teeth are the maxillary central incisors [8, 9]. Traumatic injuries to teeth and their supporting structures vary in severity ranging from enamel infraction to avulsions. The most frequent type of injury is uncomplicated crown fractures [10, 11]. These fractures may involve a single tooth or multiple teeth at the same time, the pattern of the fracture lines being a function of the direction and impact of the causative factor [8]. Root fracture is a combined injury of pulp, dentine, cementum and periodontal ligament and is considered a relatively uncommon type of dental injury [5]. The fracture line may be located at the coronal, middle or apical third of the root. Traumatized anterior teeth are often neglected and left untreated [12]. When left untreated, they tend to develop complications which may include one or a combination of the following: pulp necrosis, pulp canal obliteration, tooth discolouration, ankylosis, resorption, apical periodontitis, dento-alveolar abscess, apical granuloma or cyst [7, 13].

There are however, few reports [14] on traumatized anterior teeth in adults, especially from the developing world. Kaste et al. [14] provided estimates of the prevalence of injuries to permanent teeth in an adult representative sample of the United States (US) population. In subjects aged 21–50 years, 28.1 % showed clinical evidence of damage to the anterior dentition, with enamel fracture being the commonest injury observed. It was therefore the aim of the present study to determine the prevalence and pattern of traumatized anterior teeth among adults presenting in a tertiary health facility in Nigeria, the time interval between injury and presentation in the hospital, the reasons for seeking treatment, and the complications arising due to late presentation.

## Methods

This was a cross-sectional study carried out on adult patients presenting with traumatized anterior teeth at the University of Benin Teaching Hospital (UBTH) Dental Centre, Edo state, Nigeria, between 1st of August 2011 and 31st of May 2012. Informed consent was obtained according to ethical principles. Ethical approval was obtained from the Ethics and Research Committee of the University of Benin Teaching Hospital (ADM/F.22A/VOL.VH/370 2010).

The reliability of the data collection instrument was assessed by pre-testing and the obtained results were subjected to kappa statistics and kappa value (0.95) validated the intra-examiner variability. Clinical examination and radiographic review were carried out by one of the authors however, with regards to outcome measure, a second opinion was sought from a senior colleague and areas of discordance were reconciled.

The data collected included demographic characteristics (age, sex, occupation, level of education, and marital status). Other information obtained from history taking and clinical examination included: the when, where, and how of the injury, previous history of trauma, time elapsed between injury and presentation at the hospital, reason for seeking treatment, tooth/teeth involved, sensibility, tenderness to percussion, mobility, presence of discolouration, swelling, fistula/sinus tract, and mobility. Radiographic findings such as periapical radiolucency, pulp canal obliteration, and root resorption were also documented. Trauma to anterior teeth was classified using the Andreasen’s classification [15]. Pulp canal obliteration was diagnosed when the pulp chamber and or root canal was not discernible or reduced in size radiographically. Root resorption was diagnosed when there was blunting of the root apex resulting in a shortened root [16]. The diagnosis of pulp necrosis was based on obvious periapical radiolucency in a tooth non-responsive to electric pulp test. A retrospective diagnosis of concussion was made from patient’s history of trauma to the tooth without abnormal loosening, while subluxation was made from patient’s history of trauma to the tooth with abnormal loosening.

Data collected was analysed using the Statistical Package for Social Science (SPSS) version 16.0. The data was subjected to descriptive analysis in the form of frequencies, percentages, cross-tabulations, mean and standard deviation.

## Results

A total of 2645 adult patients attended the outpatient Department of the Dental Center at UBTH between 1st of August 2011 and 31st of May 2012. One hundred and eighty-four of them required treatment and care related to traumatized anterior teeth giving a prevalence of 6.96 %. However, 163 (88.6 %) of these patients gave consent to participate in the study. The study participants presented with 316 traumatized anterior teeth giving a patient: tooth ratio 1:1.94. The age range and mean age of the participants were 17–69 years and 30.6 ± 11.2 years respectively with majority (60.1 %) of participants aged 21–30 years. A total of 80 (49.1 %) and 83 (50.9 %) of the participants were males and females respectively, giving a male: female ratio 1:1.04. A majority (117, 71.8 %) were single and about three-quarters (120, 73.6 %) had attained tertiary education (Table 1).

**Table 1:** Demographic characteristics of the patients

Characteristics	Frequency n	Percent %
*Age (years)*		
≤20	14	8.6
21–30	98	60.1
31–40	26	16.0
41–50	7	4.3
51–60	16	9.8
61–70	2	1.2
*Gender*		
Male	80	49.1
Female	83	50.9
*Marital status*		
Single	117	71.8
Married	43	26.4
Widowed	2	1.2
Divorced	1	0.6
*Educational attainment*		
Primary	6	3.7
Secondary	37	22.7
Tertiary	120	73.6
Total	163	100.0

As shown in Table 2, falls accounted for most (26.4 %) of the trauma to anterior teeth followed by motor cycle accidents (18.4 %) and domestic accidents (12.9 %), while opening bottle cork with the teeth accounted for the least (1.2 %). Falls accounted for most trauma to anterior teeth in all age groups except 51–60 and 61–70 years age group, age group 21–30 years recorded the peak prevalence for all the etiological factors. Falls accounted for most trauma to anterior teeth in both genders. Trauma to anterior teeth from MVA, sporting activities, fights and occupational accidents were higher among males while falls, MCA, domestic accidents, CBPK and assaults were commoner among females. There was no significant association between gender and the aetiology of trauma to anterior teeth among the patients.

**Table 2:** Aetiology of trauma to anterior teeth among the patients

Aetiology	Frequency n	Percent %
Falls	43	26.4
Motor cycle accidents (MCA)	30	18.4
Domestic accidents	21	12.9
Cracking bone and palm kernel (CBPK)	15	9.2
Motor vehicle accidents (MVA)	14	8.6
Fights	13	8.0
Sporting activities	10	6.1
Assaults	10	6.1
Occupational accident	5	3.1
Opening bottle cork	2	1.2
Total	163	100.0

Assaults included cases of domestic violence, physical abuse and gunshot

**Fig. 1:**
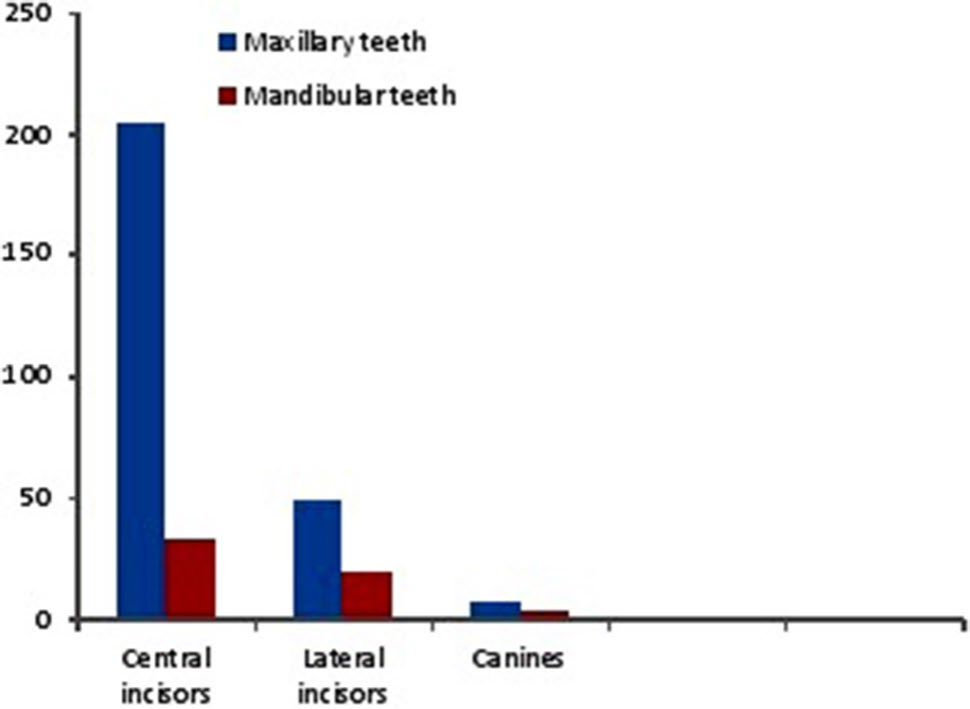
Distribution of traumatised teeth by arch

Based on Andreasen’s classification, the most common type of fracture among the patients in this study was enamel dentine fracture accounting for 28.8 %, followed by complicated crown fracture 18.6 %, and tooth avulsion 11.7 %. Complications were present in 73 (45.4 %) of the patients studied (Table 3). The maxillary central incisors were the most commonly traumatized teeth followed by the maxillary lateral incisors and canines (Fig. 1).

**Table 3:** Prevalence of complications associated with traumatised anterior teeth

Complication	Frequency n	Percent %
Present	73	44.8
Absent	90	55.2
Total	163	100.0

The mean time elapsed between injury and presentation among the patients was 6.86 ± 9.11 years. Over one third of the patients presented in the first month of injury, while close to half of the patients (48.5 %) presented within the first year. More than half (51.5 %) of the patients in the presented in the hospital more than 1 year after injury (Table 4). The majority of patients in the age groups ≤20 and 51–60 sought treatment because of pain, while the majority of those in the age groups 21–30 and 31–40 sought treatment because of aesthetic considerations (Table 4). Females reported aesthetic concerns more often than males while pain was the prevalent reason for seeking treatment in males.

**Table 4:** Time interval before presentation and reason for seeking treatment by patient’s age

	≤20 n (%)	21–30 n (%)	31–40 n (%)	41–50 n (%)	51–60 n (%)	61–70 n (%)	Total n (%)
*Time interval between injury and presentation in the hospital*							
<1 month	9 (64.3)	53 (54.1)	2 (7.7)	0 (0.0)	0 (0.0)	0 (0.0)	64 (39.3)
1–12 months	4 (28.6)	8 (8.2)	3 (11.5)	0 (0.0)	0 (0.0)	0 (0.0)	15 (9.2)
1–3 years	1 (7.1)	5 (5.1)	1 (3.8)	3 (42.9)	0 (0.0)	0 (0.0)	10 (6.1)
4–7 years	0 (0.0)	3 (3.0)	4 (15.4)	0 (0.0)	0 (0.0)	0 (0.0)	7 (4.3)
8–12 years	0 (0.0)	15 (15.3)	12 (46.2)	0 (0.0)	0 (0.0)	0 (0.0)	27 (16.6)
>12 years	0 (0.0)	14 (14.3)	4 (15.4)	4 (57.1)	16 (100.0)	2 (100.0)	40 (24.5)
Total	14 (100.0)	98 (100.0)	26 (100.0)	7 (100.0)	16 (100.0)	2 (100.0)	163 (100.0)
*Reasons for seeking treatment*							
Pain	7 (50.0)	30 (30.6)	7 (26.9)	1 (14.3)	10 (62.5)	1 (50.0)	56 (34.4)
Swelling	0 (0.0)	5 (5.1)	2 (7.7)	2 (28.6)	0 (0.0)	0 (0.0)	9 (5.5)
Aesthetic	6 (42.9)	53 (54.1)	14 (53.8)	3 (42.9)	4 (25.0)	1 (50.0)	81 (49.7)
Tooth mobility	0 (0.0)	1 (1.0)	2 (7.7)	1 (14.3)	2 (12.5)	0 (0.0)	6 (3.7)
Sensitivity	1 (7.1)	7 (7.1)	1 (3.8)	0 (0.0)	0 (0.0)	0 (0.0)	9 (5.5)
Pus discharge	0 (0.0)	2 (2.0)	0 (0.0)	0 (0.0)	0 (0.0)	0 (0.0)	2 (1.2)
Total	14 (100.0)	98 (100.0)	26 (100.0)	7 (100.0)	16 (100.0)	2 (100.0)	163 (100.0)

As shown in Table 3, seventy three (45.3 %) of the patients presented with complications involving 138 teeth. Most of the complications developed from teeth with enamel-dentine fracture. However, canal obliterations were diagnosed in teeth with concussion and subluxation injuries.

## Discussion

Epidemiological studies [17, 18] showed that the prevalence of traumatized anterior teeth varies between population and age groups. The reported prevalence of TDI was 10.9 and 12.8 % for 12-year old Nigerian school children [19, 20] and much higher (43.3 %) among adults in Finland [21]. It is not possible to diagnose all traumatized tooth after some time has elapsed from the traumatic injury since some teeth can easily fully recover and no longer be detectable from the examiner. Likewise, it is possible that a proportion of the injured have not presented for care in the hospital. Therefore, the prevalence of traumatic dental injuries to anterior teeth in the adult Nigerian population in this study of 6.96 % may not be a true reflection of the prevalence of TDI in adult Nigerians. Variation in sampling and diagnostic criteria between different studies may also explain different findings.

The age range and mean age of the patients were 17–69 years and 30.6 ± 11.2 years respectively. The association of trauma to anterior teeth with age showed a distinct pattern; rates were highest at age 21–30 years, with a marked decline by age 41–50 years. Similarly, Bastone et al. [22] reported rates highest at age 21–30 years but a slight decline by age 41–50 years. This distribution is consistent with the fact that age 21–30 years have increased physical activity and higher risk taking behavior compared to those aged 41–50 years who have relatively reduced physical activities and less risk taking behavior [23]. Unlike previous studies that have reported a marked male preponderance M:F 1.3–2.3:1 [4, 15] this study showed a slight female preponderance (M:F 1:1.04). This again may be due to the fact that the present study is a hospital based study, and that females tend to demonstrate greater interest in health and have better health seeking behavior than males [24]. This finding may also be or a pointer at increasing level of activity among females.

Similar to a previous study [4], the commonest aetiology of trauma to the anterior teeth was falls; accounting for 26.4 % of cases. This is further in agreement with studies that have found dental injuries due to falls occurring mostly during the first two decades of life [25], as majority of the patients sustained their injuries at less than 20 years of age. The second most prevalent cause of injury was motor cycle accidents (18.4 %). Although an unpopular aetiologic factor in past studies, motorcycle now feature prominently as a mode of transportation and cause of traffic accident injuries in Nigeria [26]. Among patients aged 51–60 years, cracking bone and palm kernel (CBPK) was the most common cause of traumatic injury to the anterior teeth. This is probably due to the popular dietary lifestyle among this age group. Bone meals are known for their high calcium content and the search for the “tasty” bone marrow as a delicacy or part of nutritional supplement tends to engender cracking of bone. Opening of bottle cork with the teeth accounted for 1.2 % of traumatic injuries to the anterior teeth in this study. Although this practice had been reported by Akpata [27] among school children, it is more prevalent among adults and is basically borne out of impatience, desperation and a hurried uncultured lifestyle.

The most common type of trauma using Andreasen’s classification among the patients in this study was enamel dentine fracture 91 (28.8 %), followed by complicated crown fracture 59 (18.6 %) and tooth avulsion 37 (11.7 %). This is in agreement with the study by Oluwole and Leverett [28], but is at variance with the study by Nawaf [29] in which the prevalence of enamel fracture was much higher than enamel and dentine fracture. This can be explained by the fact that those with enamel dentine fracture are more likely to have tooth sensitivity prompting them to seek treatment. The Maxillary central incisors were the most commonly traumatized teeth 205(64.8 %), followed by the maxillary lateral incisors 49(15.5 %) and Mandibular central incisors 33(10.5 %), Fig. 1. This is in agreement with several other studies [30, 31].

In the present study, slightly more than half (51.5 %) of the patients presented in the hospital more than 1 year after injury (Table 4). This may be due to lack of funds since patients pay for treatment out of pocket, unlike in the developed world where there is health insurance that takes care of treatment cost. Aesthetic concern was the most common reason for seeking treatment among the patients in the present study, this agree with the findings of Leroy et al. [32] and is in contrast with the findings of Al-Jundi’s [33] where pain or sensitivity were the main complain. This can be explained by the fact that most patients in the present study were aged 21–30 years, single and tend to be more conscious of their appearance.

Closely associated with late presentation in the hospital is the development of complications following traumatic dental injuries. Seventy three (45.3 %) of the patients in this study presented with complications involving 138 teeth. Pulp necrosis was the most common complication, followed by tooth discolouration, periapical abscess, and pulp calcification. All the teeth with pulp necrosis had apical rarefaction, were tender to percussion, and gave negative responses to electric pulp test. Hence root canal treatment was recommended. Majority of teeth with pulp canal obliteration were considered healthy and functional based on radiographic evaluation, clinical signs and symptoms, however, root canal treatment were recommended when they become tender to percussion and give negative response to sensibility test. The most dramatic tooth injuries (root fractures, intrusion and avulsions) presented 0 % complications. This is due to the fact that the patients with root fractures presented early and were treated promptly while the cases if intrusion were all extracted and the avulsed teeth were not brought to the clinic so they couldn’t be re-planted.

## Conclusions

The prevalence of TDI in this study falls within previously reported figures. However, the high number of teeth developing complications may have resulted from late presentation for care.

## Abbreviations

CBPK: cracking bone and palm kernel
MCA: motorcycle accidents
PDL: periodontal ligament
SD: standard deviation
SPSS: statistical package for social sciences
TDI: traumatic dental injury
US: United States
